# Supplementary figures and images for: Bone marrow mesenchymal stem cell-derived exosomal microRNA regulates microglial polarization
Source: PeerJ. 2023 Nov 9;11:e16359. doi: 10.7717/peerj.16359 (PMC10640847; doi:10.7717/peerj.16359)

A

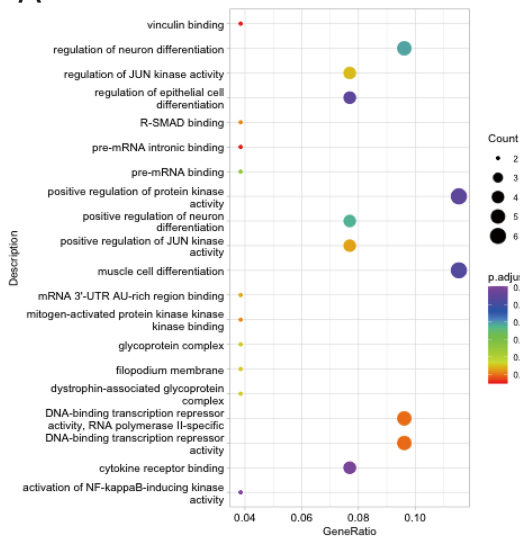

B

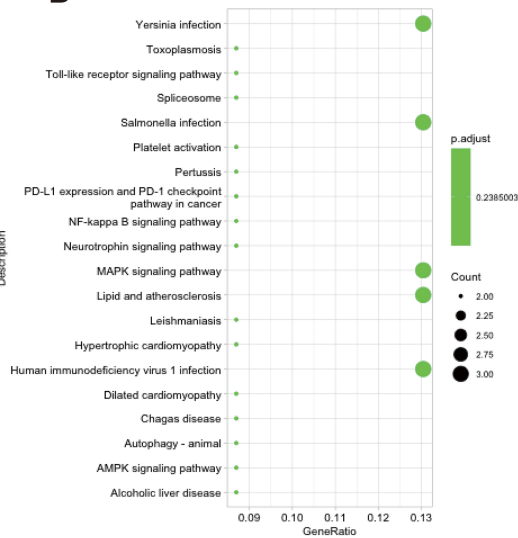

C

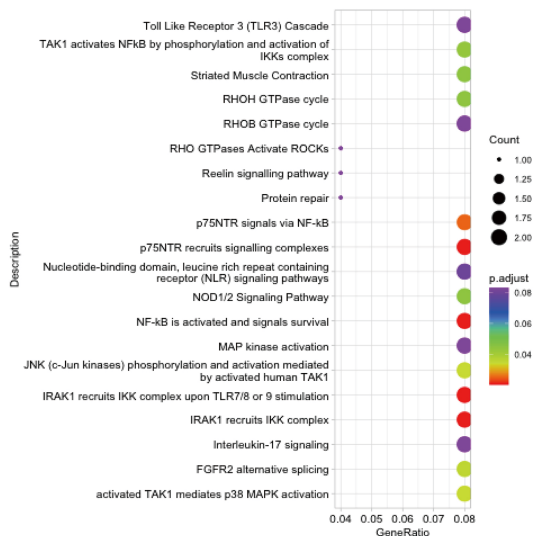

Supplement: Supplemental Information 1 — (A) Bubble plot of top 20 enriched GO terms for predicted target genes. (B) Bubble plot of top 20 enriched KEGG pathways for predicted target genes. (C) Bubble plot of top 20 enriched Reactome pathways for predicted target genes. Color represents the P value; the size of the bubble represents gene count. [file peerj-11-16359-s001.pdf]

A

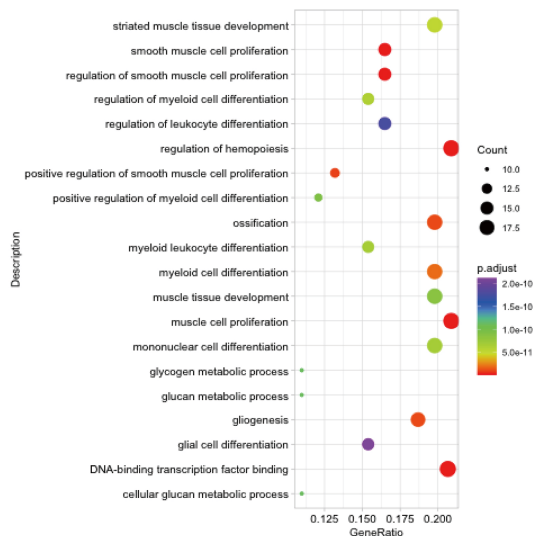

B

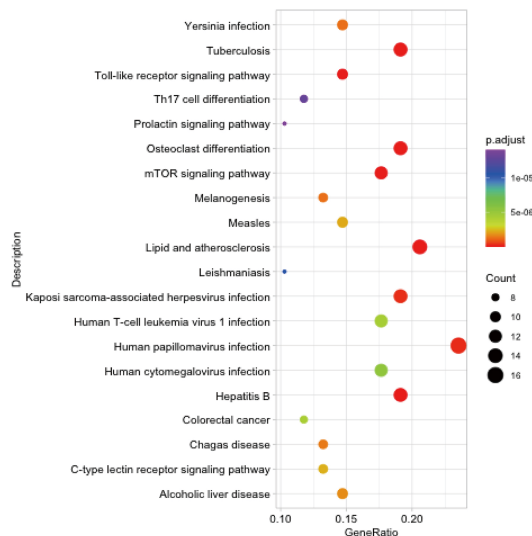

C

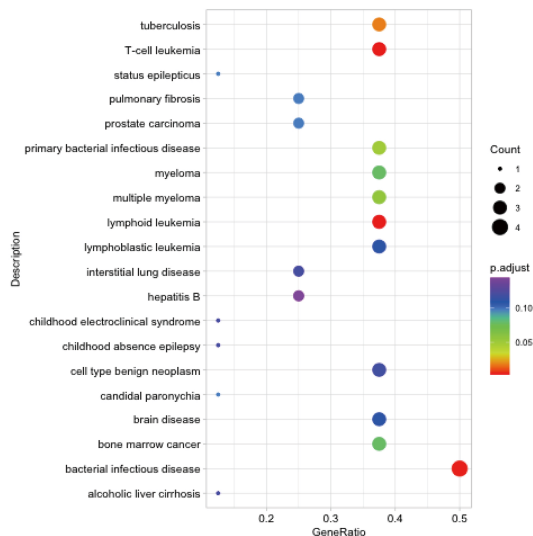

D

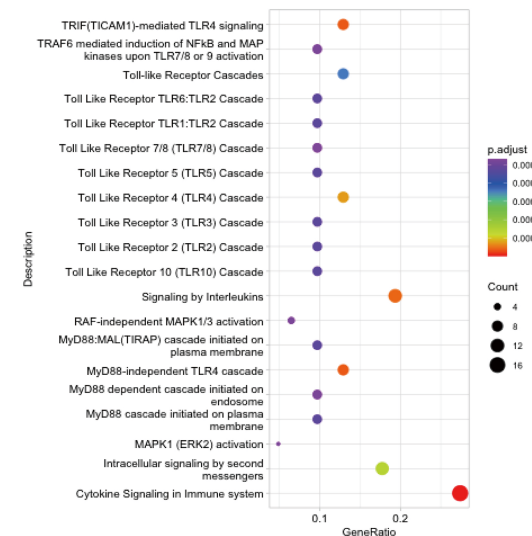

Supplement: Supplemental Information 2 — (A) Bubble plot of top 20 enriched GO terms for experimentally validated target genes. (B) Bubble plot of top 20 enriched KEGG pathways for experimentally validated target genes. (C) Bubble plot of top 20 enriched DO terms for experimentally validated target genes. (D) Bubble plot of top 20 enriched Reactome pathways for experimentally validated target genes. Color represents P-value; size of the bubble represents gene count. [file peerj-11-16359-s002.pdf]

A

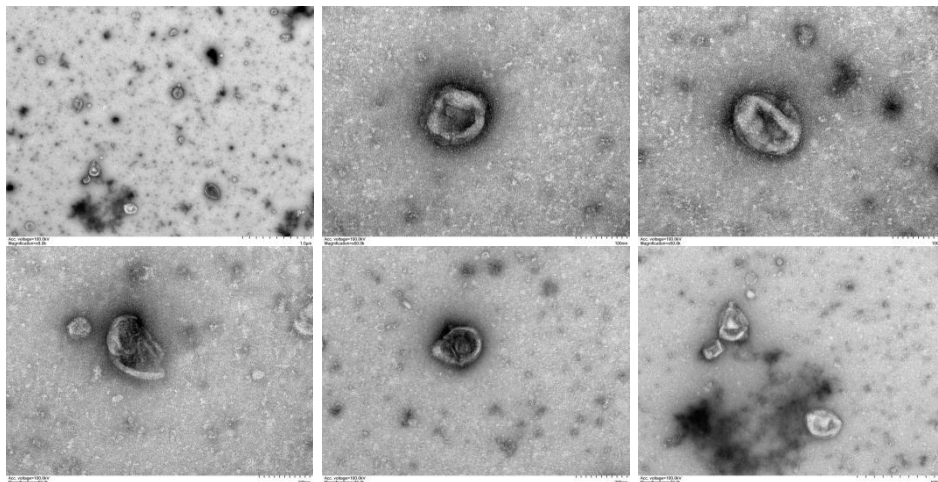

B

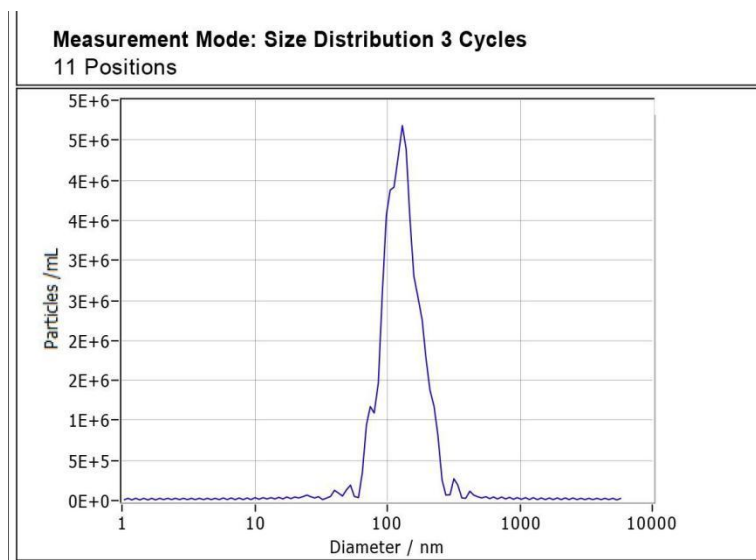

C

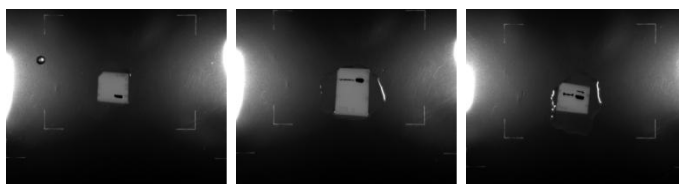

D

Control

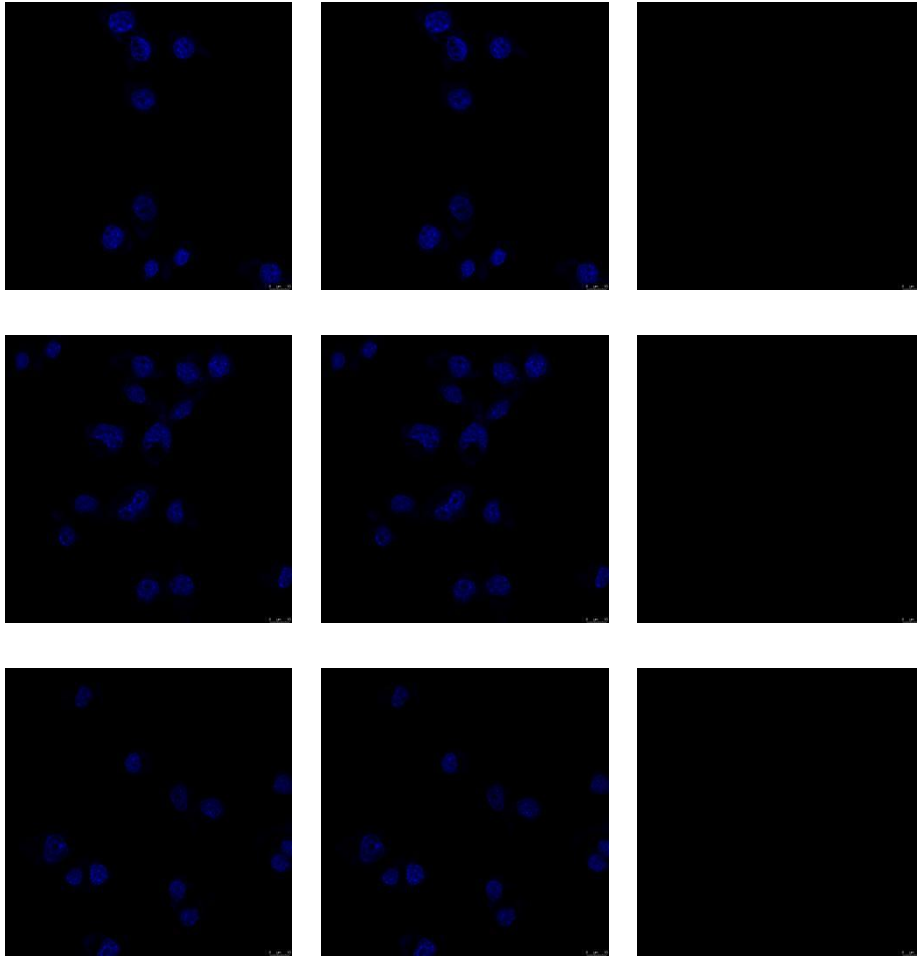

Exo

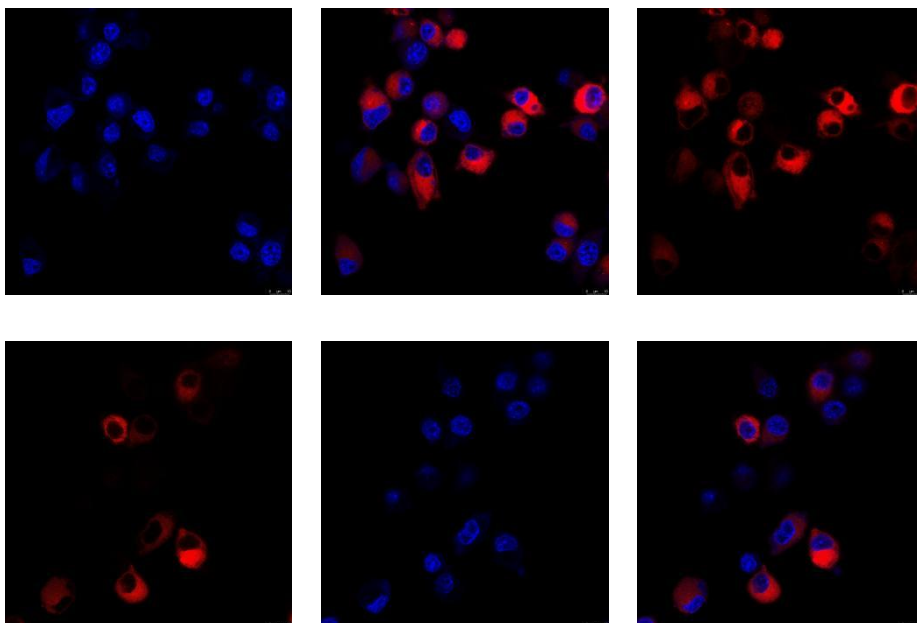

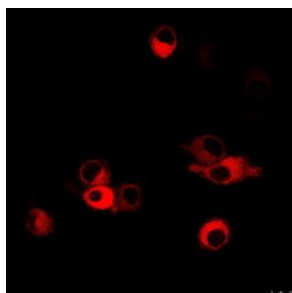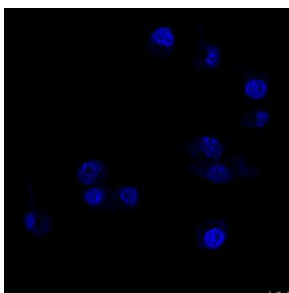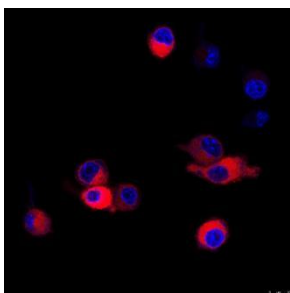

Supplement: Supplemental Information 6 [file peerj-11-16359-s006.zip › raw data/Figure 1.pdf]

1C

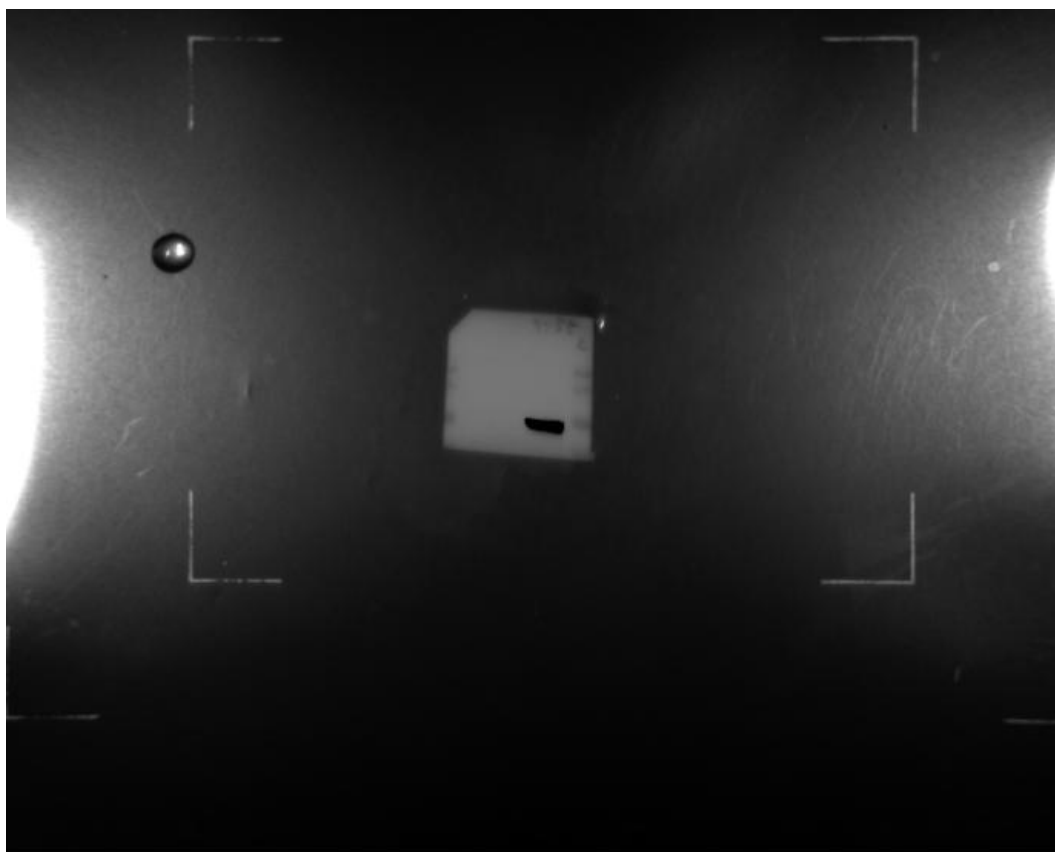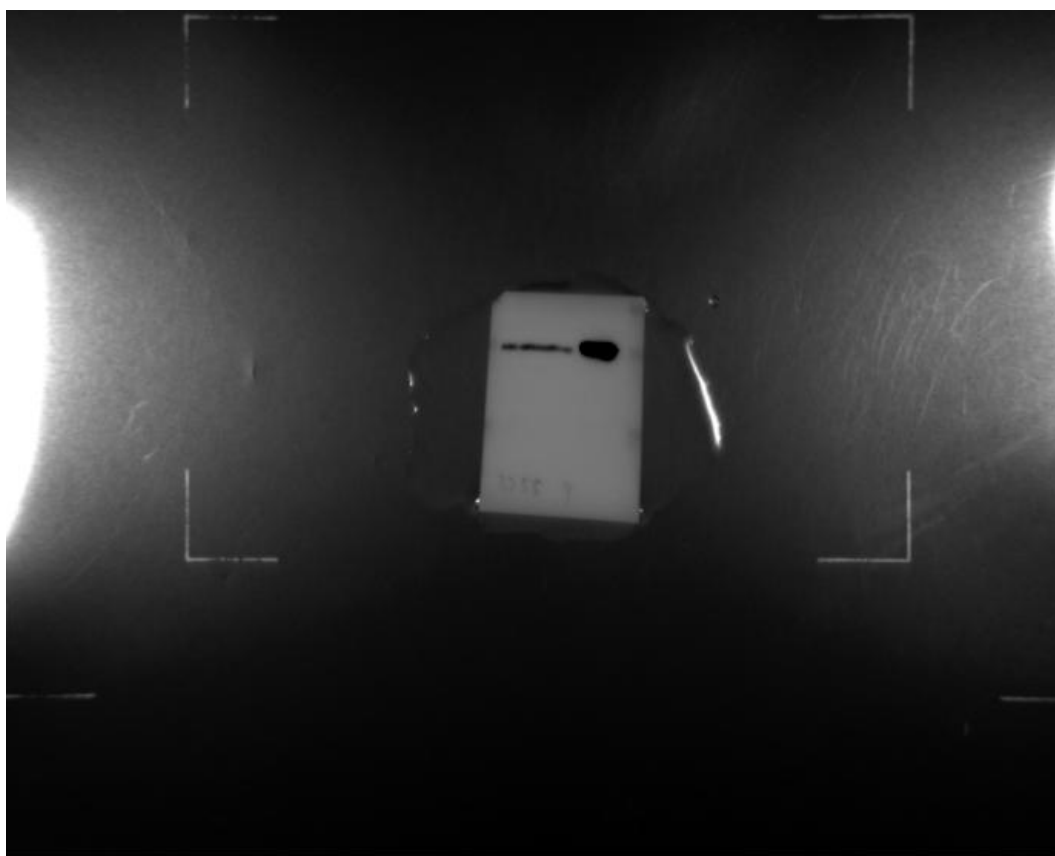

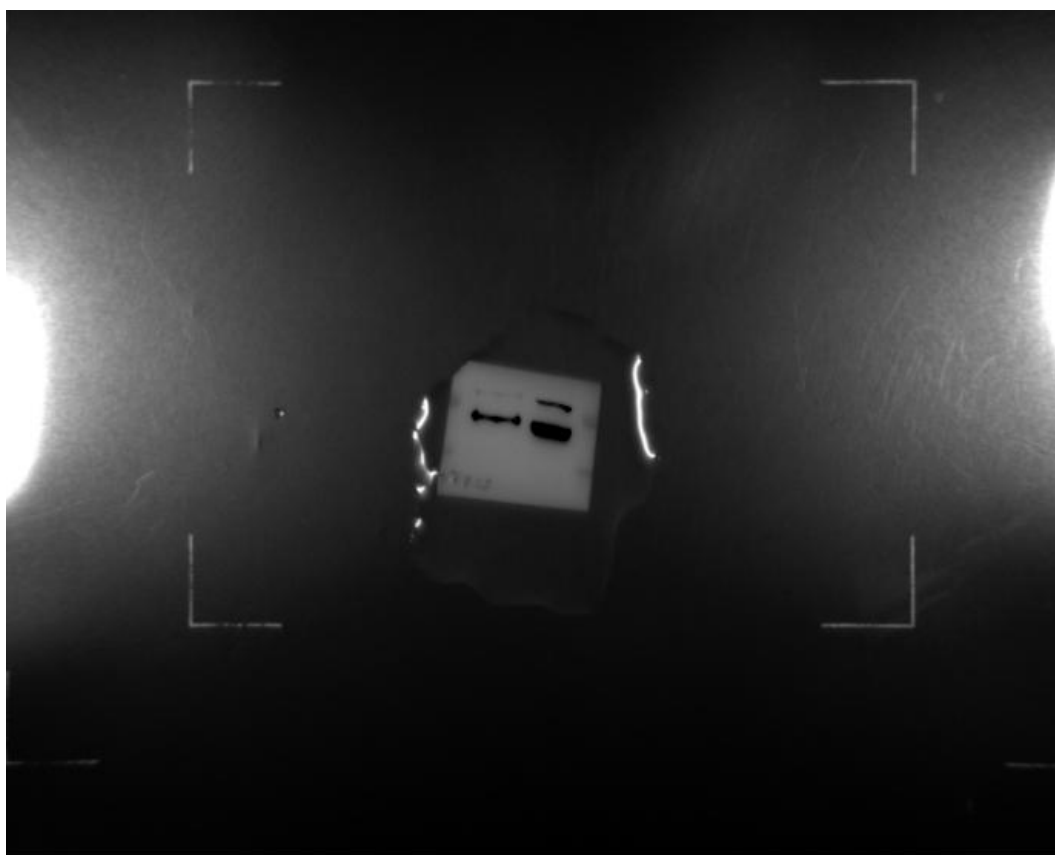

7C

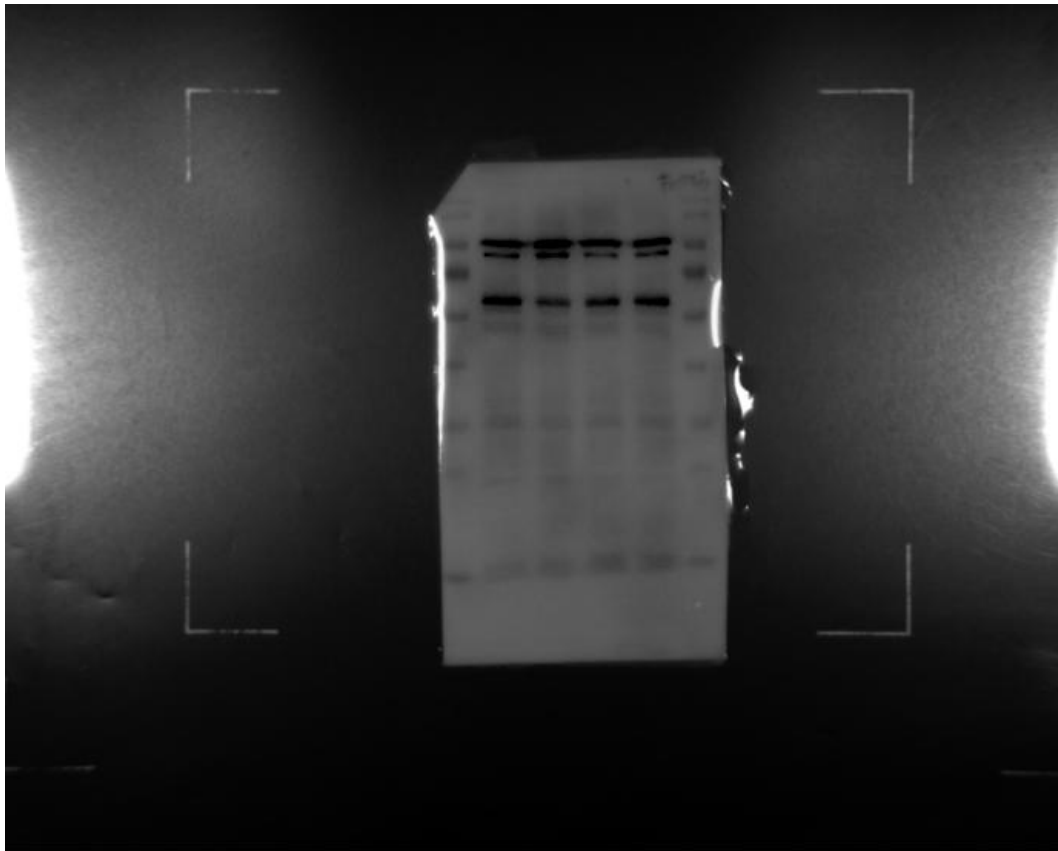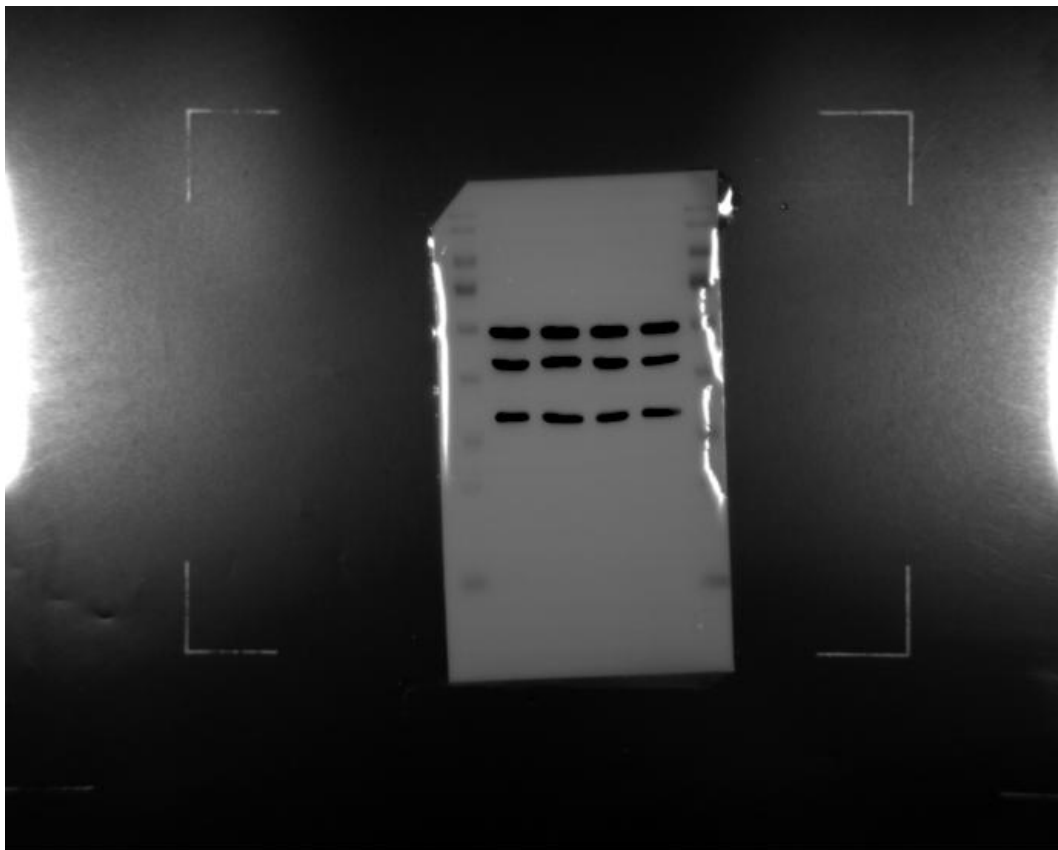

Supplement: Supplemental Information 7 [file peerj-11-16359-s007.pdf]
