# Supplementary material for: Bone marrow mesenchymal stem cell-derived exosomal microRNA regulates microglial polarization
Source: PeerJ. 2023 Nov 9;11:e16359. doi: 10.7717/peerj.16359 (PMC10640847; doi:10.7717/peerj.16359)
Supplement: Supplemental Information 3 [file peerj-11-16359-s003.docx]

Table S1 Primers in RT-qPCR

| Primers | Sequences |
| --- | --- |
| miR qRT R | CAGTGCAGGGTCCGAGGTAT |
| U6 qRT F | CTCGCTTCGGCAGCACA |
| U6 qRT R | AACGCTTCACGAATTTGCGT |
| mmu-miR-6238 qRT F | GCCGAGTTATTAGTCAGTGGAG |
| mmu-miR-3102-3p qRT F | GAGCACCCCATTGGCTA |
| mmu-miR-6984-5p qRT F | ACTGAAAGGCAATGAAGG |
| mmu-miR-495-3p qRT F | GCCGAGAAACAAACATGGTGCA |
| mmu-miR-3095-3p qRT F | TGGACACTGGAGAGAGA |
| mmu-miR-6975-5p qRT F | GCTGGGGAGAAAGGGGT |
| mmu-miR-7013-5p qRT F | TATGAAGAGGCCAGTG |
| mmu-miR-7652-5p qRT F | TAAGGGCACAGGGATT |
| mmu-miR-146a-5p qRT F | agcccgTGAGAACTGAATTCCA |

R: reverse; F: forward.
