# Supplementary material for: Bone marrow mesenchymal stem cell-derived exosomal microRNA regulates microglial polarization
Source: PeerJ. 2023 Nov 9;11:e16359. doi: 10.7717/peerj.16359 (PMC10640847; doi:10.7717/peerj.16359)

A

Mimics NC

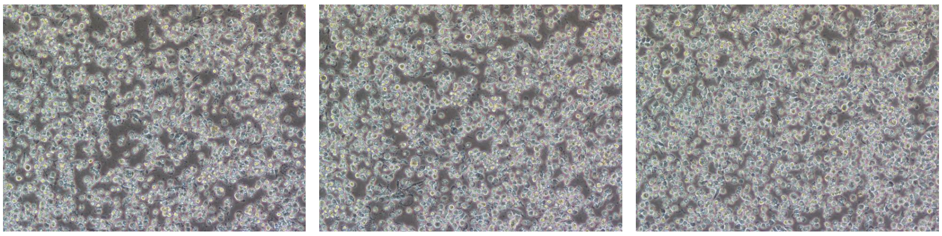

Mimics

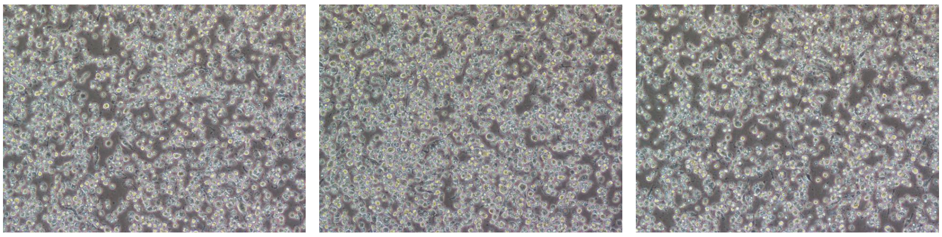

Inhibitor NC

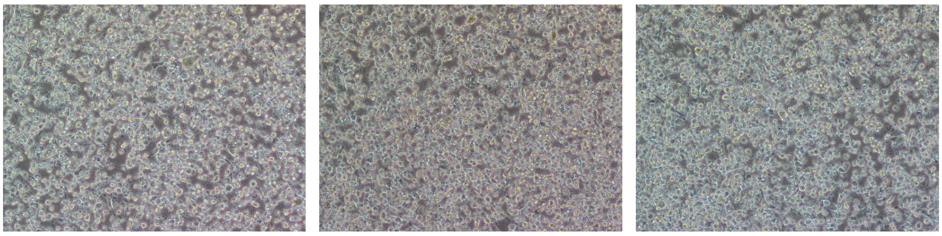

Inhibitor

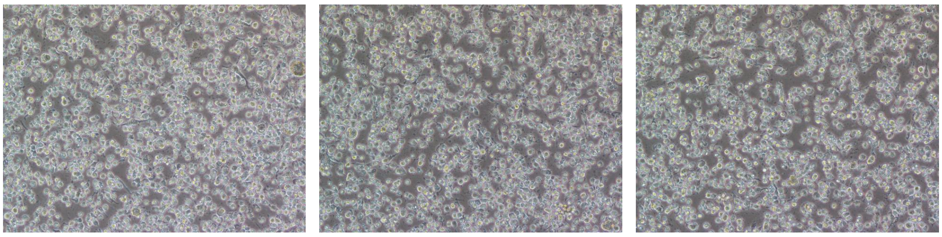

B

CD40

Mimics NC

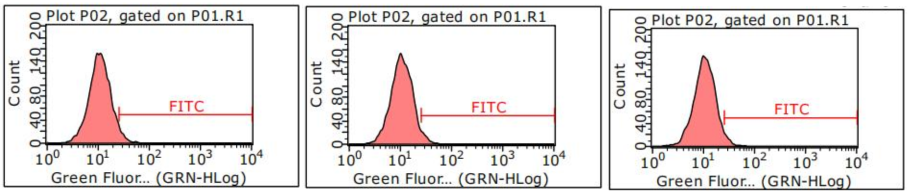

Mimics

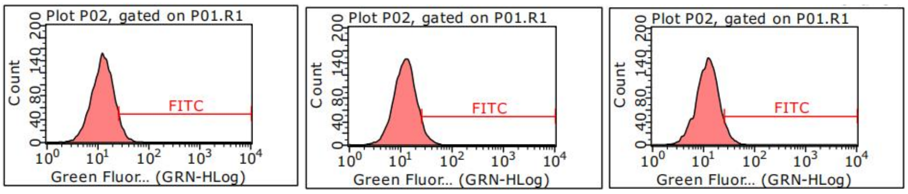

Inhibitor NC

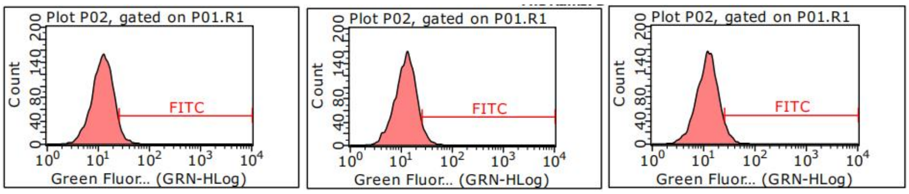

Inhibitor

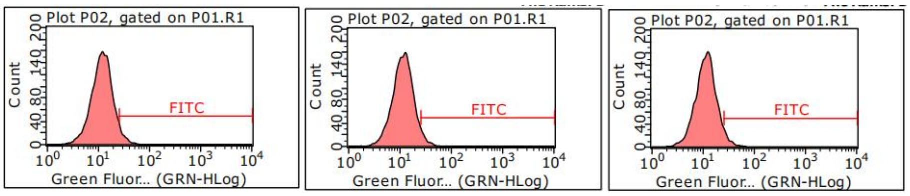

CD86

Mimics NC

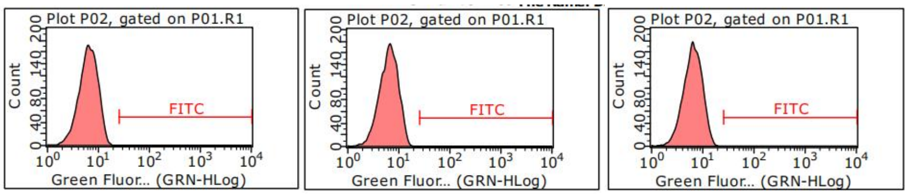

Mimics

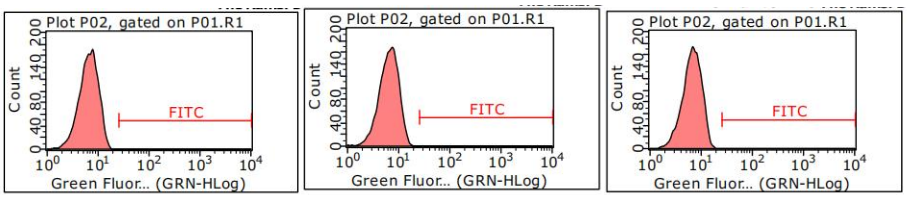

Inhibitor NC

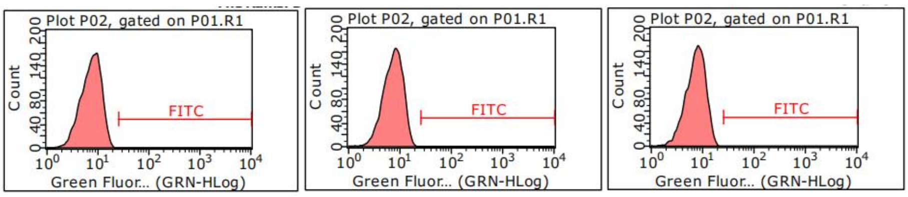

Inhibitor

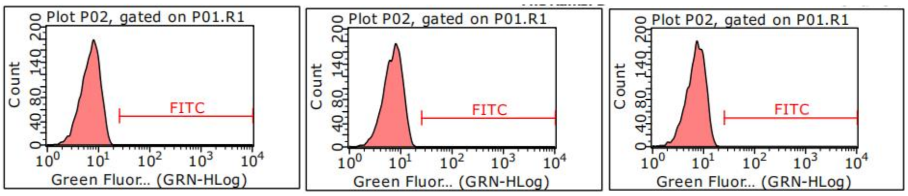

CD206

Mimics NC

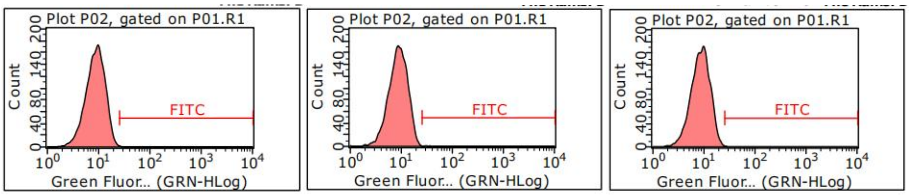

Mimics

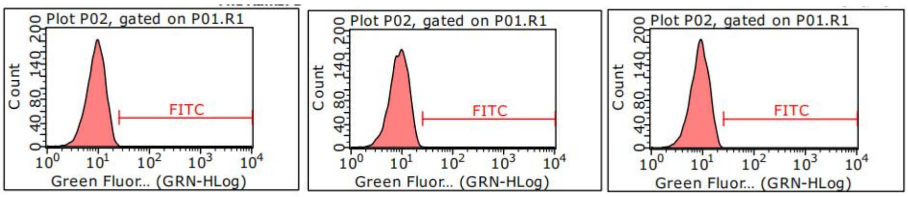

Inhibitor NC

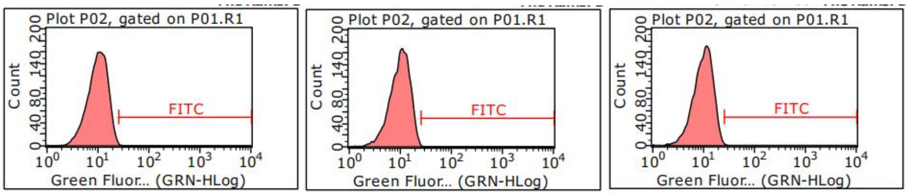

Inhibitor

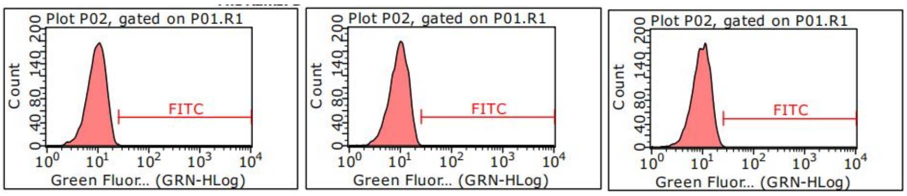

Supplement: Supplemental Information 6 [file peerj-11-16359-s006.zip › raw data/Figure 6.pdf]
