# Supplementary material for: Bone marrow mesenchymal stem cell-derived exosomal microRNA regulates microglial polarization
Source: PeerJ. 2023 Nov 9;11:e16359. doi: 10.7717/peerj.16359 (PMC10640847; doi:10.7717/peerj.16359)

C

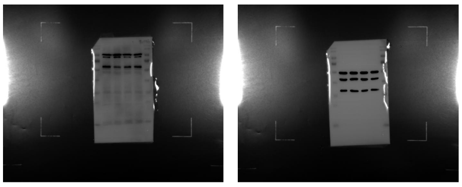

D

Control

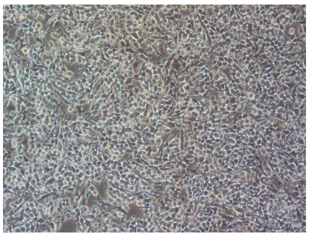

Sh Traf6-1

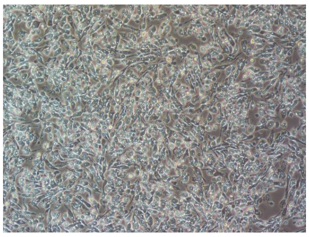

Sh Traf5-2

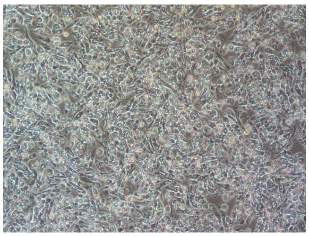

Sh Traf6-3

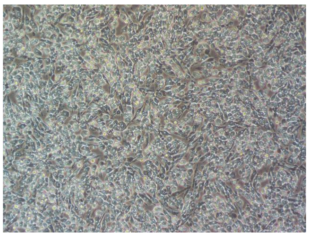

F

Mimics + oe-NC

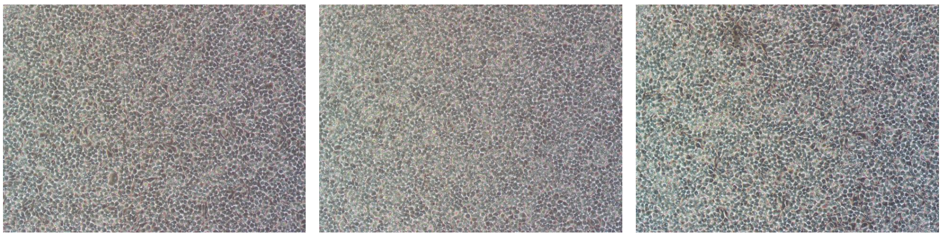

Mimics + oe-Traf6

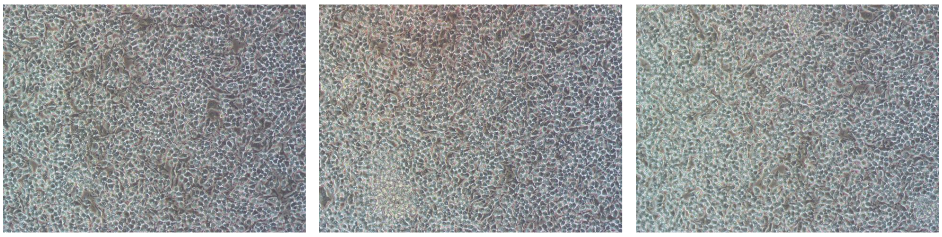

Inhibitor + sh-NC

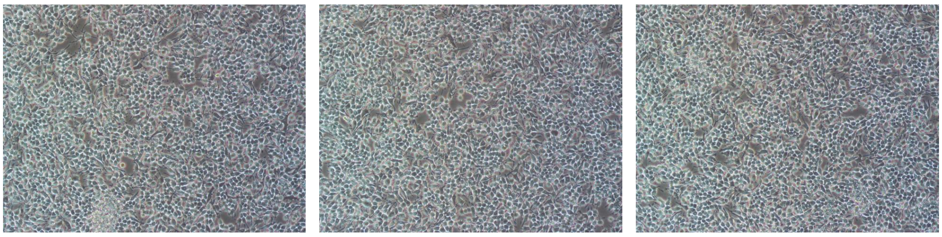

Inhibitor + shTraf6-1

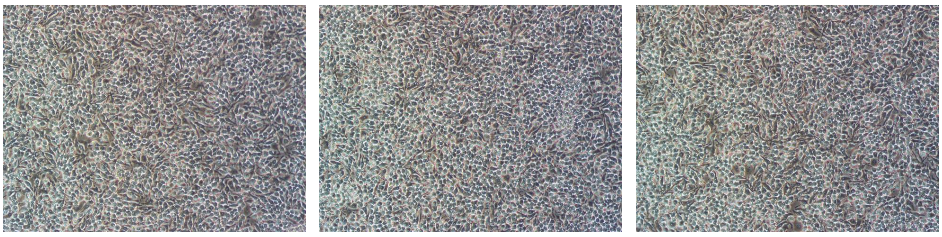

Inhibitor + shTraf6-2

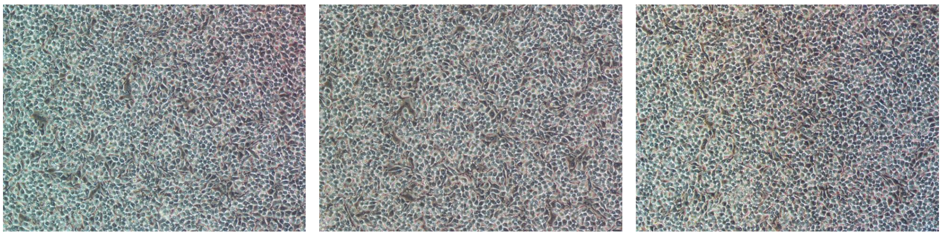

Supplement: Supplemental Information 6 [file peerj-11-16359-s006.zip › raw data/Figure 7.pdf]
