# Supplementary material for: Bone marrow mesenchymal stem cell-derived exosomal microRNA regulates microglial polarization
Source: PeerJ. 2023 Nov 9;11:e16359. doi: 10.7717/peerj.16359 (PMC10640847; doi:10.7717/peerj.16359)
Supplement: Supplemental Information 8 [file peerj-11-16359-s008.docx]

**Checklist for Quantitative PCR Assays**

1. **Sample**
   - Fresh - How rapidly processed?
   - Immediately extract RNA after collection sample
   - Frozen - How frozen?
   - Whole vs. microdissected
   - Sample storage conditions and duration
   - Fixed - How fixed, how old?
2. **Nucleic acid**
   - Quantification
   - Use Nano-Drop 8000 to measure concentration
   - Quality/integrity
   - Evaluation of RNA quality by RNA agarose gel electrophoresis
   - Inhibition dilution or spike
   - No inhibitors used
   - DNA contamination assessment of RNA sample
   - A_260_/A_280_=1.95
   - DNase treatment
   - Manufacturer of reagents used
   - Tripure（Roche）
   - Amount of sample used for extraction
   - Cell：10^6^
3. **Reverse treanscriptions**
   - cDNA priming method + concentration
   - Amount of RNA used per reaction
   - 2 μg
   - Enzyme type and concentration
   - Detailed reaction conditions
   - 65 ℃，5 min；25 ℃孵育5 min；42 ℃，60 min；70 ℃，5 min
   - Manufacturer of reagents used
   - RevertAid First Strand cDNA Synthesis Kit（Invitrogen）
   - Reaction volume
   - 20 μL
   - Storage of cDNA
   - -80℃
4. **Target**
   - Database name and target gene accession number
   - Intronless, targeting of all splice variants/splice variant-specific targeting
   - Official gene symbol
   - Location of amplicon with respect to reference sequence
   - Information about (retro)pseudogenes
5. **Primers and probes**
   - Primer sequences

| Genes | Primer sequences |
| --- | --- |
| U6 qRT F | CTCGCTTCGGCAGCACA |
| U6 qRT R | AACGCTTCACGAATTTGCGT |
| mmu-miR-6238 qRT F | GCCGAGTTATTAGTCAGTGGAG |
| mmu-miR-3102-3p qRT F | GAGCACCCCATTGGCTA |
| mmu-miR-6984-5p qRT F | ACTGAAAGGCAATGAAGG |
| mmu-miR-495-3p qRT F | GCCGAGAAACAAACATGGTGCA |
| mmu-miR-3095-3p qRT F | TGGACACTGGAGAGAGA |
| mmu-miR-6975-5p qRT F | GCTGGGGAGAAAGGGGT |

- - Location of modification
  - 5' region
  - End concentration of primers and optional probe(s) used
  - 10 μM
  - Primer purification method
  - Manufacturer of oligonucleotides
  - Probe sequence

1. **Assay details**
   - Amplicon length
   - Specific BLAST or equivalent in silico specific screen
   - Experimental validation of specificity
   - NTC; Sensitivity
   - PCR efficiency, PCR efficiency standard curve slope and r-squared value
   - RTPrimerDB ID
   - Secondary structure analysis around priming sites
2. **PCR Cycling**
   - Amount of cDNA/DNA used per reaction
   - 8 μL
   - Detailed reaction conditions, thermocycling parameters
   - 95 ℃，10 min；95 ℃，15 s；60 ℃，20 s；72 ℃，25 s；72 ℃，5 min；40 cycles
   - Manufacturer of reagents used
   - SYBR Green qPCR Master（Roche）
   - Manual/robotic dispensing of reagents
   - Manufacturer of plates/tubes
   - Manufacturer of real-time instrument
   - Bio-rad
3. **Data analysis**
   - Cq value determination method
   - The number of amplification cycles that occur during qPCR amplification when the fluorescence signal of the amplification product reaches the set threshold.The number of amplification cycles is linearly proportional to the logarithm of the initial concentration.
   - Treatment of NTCs and technical replicates
   - Normalisation method
   - linear normalization
   - Is r-squared value of regression curve satisfactory?
   - R2>0.98
   - Has assay sensitivity been adequately evaluated and described?
   - Has assay specificity been adequately described?
   - Judging by whether the dissolution curve is a single peak
   - Is the dynamic range of the assay acceptable?
   - Is the coefficient of variation for inter and intra-assay reproducibility reasonable?
   - Concordance of biological replicates
   - Analysis program
   - GraphPad
   - Assay carried out by core lab or investigator's lab
   - Acknowledgement of author's contribution to analysis and interpretation
   - Submission of Cq values of raw data using RDML
